# Exploring Disparities in Opioid Utilization: An Analysis Comparing Red River Métis to All Other Manitobans

**DOI:** 10.23889/ijpds.v9i5.1668

**Published:** 2024-09-18

**Authors:** Kyler Nault, Nathan Nickel, Colton Poitras, Frances Chartrand, Michelle Driedger, Alan Katz, Olena Kloss

**Affiliations:** 1 Manitoba Metis Federation; 2 University of Manitoba, Department of Community Health Sciences; 3 Manitoba Center for Health Policy

## Objective and Approach

To address the opioid crisis within the Red River Métis (RRM) Community, understanding opioid use is crucial to empower regional health authorities to adapt health programs, services, and policies to meet their unique needs effectively.

The investigation utilized focus groups with a Community-Based Participatory Research and Collective Consensual Data Analytics Procedure (CBPR/CCDAP) approach. Additionally, a population-based retrospective cross-sectional study for fiscal years 2006/07–2018/19 was conducted using administrative data from a population research data repository. Rates of prescription opioid dispensing (RPOD) and mean morphine equivalents (MEQ) were compared between RRM and all other Manitobans (AOM) aged 10 years or older.

## Results

The rate of prescription opioid dispensing and MEQ/person were found to be consistently higher among RRM compared to AOM in each study year (p < 0.001). While the RPOD declined among AOM over the study period, it did not change among RRM. Key findings revealed RRM were concerned about how opioids impacted their communities, and felt the need for increased addiction treatment resources, including Red River Métis culture-specific programs.

## Conclusion

The evidence demonstrates higher RPOD and MEQ among RRM compared to AOM, suggesting elevated risk of opioid-related harms. Focus group feedback reinforces the need for tailored interventions.

## Implications

Future policies and programs targeting the opioid crisis should prioritize the unique needs of populations like the RRM, requiring tailored, culturally appropriate interventions for effective crisis management.

